# Radiology- and gene-based risk stratification in small renal cell carcinoma: A preliminary study

**DOI:** 10.1371/journal.pone.0256471

**Published:** 2021-09-07

**Authors:** Seiichiro Takao, Yasuhiro Ushijima, Yushi Motomura, Katsumi Sakamoto, Masakazu Hirakawa, Akihiro Nishie, Koshi Mimori, Yasuo Yamashita, Takashi Tsutsumi, Kousei Ishigami

**Affiliations:** 1 Department of Radiology, Beppu Hospital, Kyushu University, Beppu, Japan; 2 Department of Clinical Radiology, Graduate School of Medical Sciences, Kyushu University, Fukuoka, Japan; 3 Department of Advanced Imaging and Interventional Radiology, Graduate School of Medical Sciences, Kyushu University, Fukuoka, Japan; 4 Department of Surgery, Beppu Hospital, Kyushu University, Beppu, Japan; 5 Department of Medical Technology, Kyushu University Hospital, Fukuoka, Japan; 6 Canon Medical Systems Corporation, Tochigi, Japan; University of California San Francisco, UNITED STATES

## Abstract

**Purpose:**

Most small renal cell carcinomas (small RCCs) will remain indolent after detection, but some stage I RCCs still metastasize. There are no risk-stratification imaging factors that could be used to identify poor-prognosis patients based on genomic profiling. Here, we evaluated the relationships between imaging parameters and RNA expressions in small RCC and attempted to identify imaging factors that could be used as effective biomarkers.

**Methods:**

We acquired biopsy specimens of 18 clear cell carcinomas that had undergone perfusion CT (pCT) and MRI between April 2018 and March 2019. We performed RNA sequencing, assessed RNA expressions, and calculated each tumor’s cell-cycle progression (CCP) score, which has prognostic value in predicting metastatic progression. We classified the tumors into two groups: clear cell type A (ccA) and type B (ccB). CcA has better survival compared to ccB. We evaluated the following characteristics of each tumor: tumor size, presence of pseudocapsule, and fat. We used the pCT and MRI to measure each tumor’s volume transfer constant (Ktrans), rate constant (Kep), extracellular extravascular volume fraction (VE), fractional plasma volume (VP), and apparent diffusion coefficient (ADC). The correlations between these small RCC imaging parameters and the tumor size and RNA expressions were determined.

**Results:**

The tumor size was significantly correlated with Kep and inversely correlated with VE, VP, ADC, and hallmark angiogenesis. The CCP score was significantly inversely correlated with Ktrans and Kep. The ccA tumors tended to show a pseudocapsule on MRI.

**Conclusion:**

Tumor size was correlated with low perfusion, but not with prognostic factors based on genomic profiling. Imaging parameters (e.g., Ktrans and Kep) and tumor characteristics (e.g., pseudocapsule) may enable gene-based risk stratification in small RCC.

## Introduction

The increase in the renal cell carcinoma (RCC) incidence over the past three decades is attributed to the increased use of cross-sectional imaging [1]. The rising incidence of RCC is largely accounted for by small (≤4 cm) tumors, which are diagnosed as stage I; most of these tumors are asymptomatic at diagnosis [2]. The clinical outcomes of small renal cell carcinoma (small RCC) patients is generally excellent, but ~25% of high-grade stage I RCCs metastasize [3]. The identification of risk stratification factors in small RCC that can be used to predict poor prognoses is thus desired.

Tumor size contributes to RCC risk stratification; e.g., for the identification of appropriate tumors for active surveillance (AS), which is defined as the initial monitoring of a tumor’s size by abdominal imaging techniques such as computed tomography (CT) and magnetic resonance imaging (MRI) [4], with delayed intervention when indicated. There are no universally accepted guidelines for determining the need for upfront or delayed intervention in RCC cases [4]. This suggests that tumor size does not provide an accurate indicator of malignancy, such as progression and metastasis.

Core biopsies underestimate the nuclear grade [5]. A standard biopsy plus the incorporation of genomic profiling could be useful for stratifying RCC patients into high-, intermediate-, and low-risk subgroups [6], as the introduction of cancer genomics to clinical practice has revolutionized the diagnostic, prognostic, and therapeutic approaches to various malignancies. Although the biopsy + genomic profiling approach is now technically feasible, its high cost and the potential complications posed by a biopsy may prevent its widespread adoption [7].

Radiology modalities such as CT and MRI play vital roles tumor surveillance. They can be used to obtain the precise tumor diameter, and image findings such as the identification of fat or a pseudocapsule and imaging parameters (e.g., the apparent diffusion coefficient [ADC]) could be associated with the tumor grade and recurrence as well as the differential diagnoses of renal tumors [8]. CT and MRI are noninvasive and low-cost compared to a genomic analysis. New functional imaging techniques such as perfusion CT (pCT) provide molecular-level findings and new perspectives in small RCC imaging. For example, pCT parameters are reported to be useful for histological diagnoses and for assessing the response to anti-angiogenic therapy [9].

We speculated that the latest radiological imaging techniques combined with genomic profiling may identify new biomarkers beyond tumor size for small RCC surveillance. Herein, we evaluated the associations between imaging parameters and gene expressions in small RCC and identified imaging factors that may be effective biomarkers.

## Materials and methods

### Patients and sample collection

**Fig 1:**
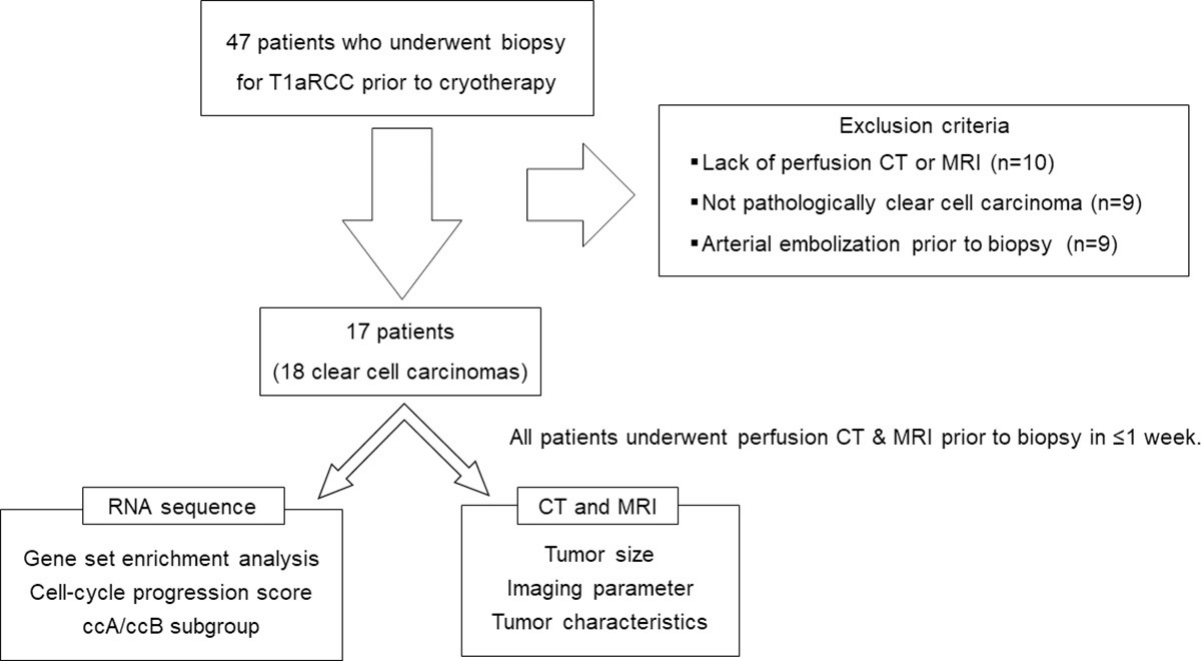
The study protocol.

This study was approved by the Kyushu University Ethics and Indications Committee (no. 30–181). Written informed consent was obtained from all participants and there were no pediatric patients among them. Small RCC samples were obtained from 47 patients who underwent cryotherapy for the primary tumor at our institution from April 2018 to March 2019. A preoperative biopsy with an 18-ga. core needle was performed in all patients. No patients received preoperative chemotherapy. Patients were excluded for the following: lack of pCT or MRI data (n = 10), difficulty in diagnosing clear cell carcinoma (n = 9), and cases of arterial embolization prior to biopsy (n = 9). Seventeen patients (18 tumors) were enrolled: 14 men, three women (ages 52–88, mean age 72 years, Table 1). All patients underwent pCT and MRI within 1 week before their biopsy (Fig 1).

**Table 1:** The characteristics of the 17 patients with small renal cell carcinoma (small RCC).

**Clinical characteristics:**	
Age, yrs	76 (63–81.75)
Males/females	13 (76%)/4 (24%)
BW, kg	68.75 (59–76.1)
Height, cm	163.85 (157–169.2)
BMI, kg/m^2^	25.89 (23.54–27.17)
Cr, mg/dL	0.93 (0.81–1.14)
eGFR, mL/min/1.73m^2^	62 (47.5–72)
**Tumor characteristics:**	
Tumor dia., mm	24 (15.7–34.2)
Presence of fat	7 (39%)
Recognition of a pseudocapsule	12 (67%)

The data are median (interquartile range) or n (%). BMI: body mass index, BW: body weight, Cr: creatine, eGFR: estimated glomerular filtration rate.

### Tissue collection and RNA extraction

All biopsy samples were immediately placed in RNA Later (Takara, Tokyo) and stored at −80°C until RNA extraction. Total RNA was extracted from each sample using ISOGEN-II (Nippon Gene, Toyama, Japan) per the manufacturer’s instructions as described [10].

### RNA sequencing and data analysis

RNA extracted from tissues was sequenced using the desktop sequencer BGISEQ-500 (BGI, Beijing). RNA-seq reads were obtained in fastq file format. The reads were aligned to the human reference sequence and gene annotations (UCSC hg19) using Tophat2 ver.2.0.14 [11]. STAR ver.2.5.2a [12] was used to calculate fragments per kilobase of transcript per million fragments mapped (FPKM) values. A differential expression analysis was performed using DESeq2 [13]. A gene ontology (GO) analysis was performed using topGO [14]. The obtained sequence data were normalized with quantile normalization [15].

### Evaluating transcriptomic signatures for RCC risk stratification

Multiple transcriptomic signatures have been investigated to risk-stratify clear cell carcinoma [16]. We selected signatures that were: (1) adaptable to RNA sequence data and (2) tested in studies other than the original study. We eventually decided to evaluate the RCCs with two assays: the cell-cycle progression (CCP) score and the division of the tumors into the clear cell type A (ccA) and clear cell type B (ccB) subgroups. The CCP score was calculated as the mean of the normalized counts for the 31 CCP genes [17]. It has been reported that the CCP score has prognostic value in predicting metastatic progression [17] and is also a significant, independent predictor of long-term oncologic outcomes in RCC [18]. For the designation of ccA or ccB, we applied k-means clustering to the normalized sequence data based on earlier investigations [19–21]. The ccA and ccB subtypes are RCC types clustered by RNA expression [20, 21], and RCCs classified as ccA have a better prognosis than those classified as ccB. We also evaluated the relationship between the tumor size and variation in previously defined gene sets by conducting a gene set enrichment analysis (GSEA). The biologically defined gene sets were obtained from the Molecular Signatures Database ver. 5.2 (http://software.broadinstitute.org/gsea/msigdb/index.jsp). We chose 50 hallmark gene sets for the GSEA because they were generated by a computational methodology based on identifying gene set overlaps and retaining genes that display coordinate expression. The hallmarks reduce noise and redundancy and provide a better delineated biological space for a GSEA [22].

### Perfusion CT imaging protocol

All patients were examined with a 320-detector CT scanner (Aquilion ONE ViSION Edition, Canon Medical Systems, Otawara, Japan) at precontrast and at 27 timepoints from 10 to 124 sec after the start of an injection of nonionic contrast agent (Iopamiron 370 mg/mL; Bayer Healthcare, Osaka, Japan) using a power injector. To reduce respiratory artifacts, the patients were instructed to breathe gently during the scan acquisition. Table 2 lists the scan parameters. An unenhanced CT scan of the upper abdomen covering the kidneys was performed first to locate the renal lesion. A supervising radiologist identified the tumor and placed the predefined scan volume. Immediately after pCT scans, a conventional contrast-enhanced CT examination of the abdomen and thorax was performed.

**Table 2:** Details of the perfusion CT and MR sequence parameters.

**320-detector CT:**				
Scan range, mm		160		
Tube voltage, kV		100		
Tube current, mA		90		
Rotation time, sec/rot		0.5		
Collimation, mm		0.5 × 320		
Matrix, pixels		512 × 512		
Field of view, mm		320		
Reconstruction thickness, mm		1		
Reconstruction kernel		FC13		
Reconstruction method		Adaptive iterative dose reduction (AIDR 3D, Standard/Strong)		
Volume CT dose index, mGy		42		
Dose-length product, mGy-cm		672		
Median effective dose, mSv		10.1		
Contrast injection rate		40 mL; 5 mL/min, followed by 40 mL of saline		
**3.0 T MRI:**				
**Parameters**	**Fast spin-echo T2-weighted images**		**Dual-echo FFE giving both in-phase and out-of-phase T1-weighted images**	**DWI**
Repetition time, ms	1116		130	1469
Echo time, ms	75		1.15/2.3	52
Flip angle, degree	90		60	90
Field of view, mm	380 × 302		360 × 323	380 × 380
Matrix (frequency × phase), mm	400 × 169		272 × 195	112 ×167
Slice thickness, mm	5.0		5.0	5.0
Slice gap	1.0		1.0	1.0
No. of slices	25		25	25
No. of excitations	2		1	1
Half scan factor	0.800		N/A	0.671
Fat suppression	SPIR		N/A	SPIR
SENSE factor	2		2	2.5
K-space ordering	Linear		Linear	Linear
TSE factor	17		N/A	N/A
EPI factor	N/A		N/A	67
B-factors, sec/mm^2^	N/A		N/A	0, 500 and 1,000
Diffusion gradients	N/A		N/A	3 axes
Respiratory control	Respiratory triggered		Breath-hold	Respiratory triggered
Band width Hz/pixel	440.1		1276.6	30.9
Scan time of whole slices, min:sec	01:48		00:13.3	01:48

DWI: diffusion-weighted imaging, EPI: echo-planar imaging, FFE: fast field echo, N/A: not applicable, SENSE: sensitivity encoding

SPIR: spectral presaturation with inversion recovery.

The pCT data were post-processed into maps of the permeability parameters (Ktrans: volume transfer constant, Kep: rate constant, VE: extracellular extravascular volume fraction, VP: fractional plasma volume) as described [23]. To reduce motion effects, image registration (Body Registration software, Otawara, Japan) was performed first. The 24 image datasets were post-processed using CT permeability software (Olea Sphere3.0, Olea Medical, La Ciotat, France) by an extended tofts model as follows. The perfusion maps were generated by CT permeability software, using 3-mm-thick sections. To optimize the soft tissues’ visualization, a processing threshold (CT value range) between –30 and 400 Hounsfield units (HU) was used, and the analysis matrix and noise elimination level were set at 128 and strong, respectively. An arterial input was defined within the renal artery by using a mouse to place a circular region-of-interest (ROI). The Ktrans, Kep, VE, and VP values were calculated, and the perfusion maps were generated.

### MRI imaging technique

MR imaging was performed using a 3.0 T MRI unit (Ingenia, Phillips Healthcare, Best, Netherlands) with a 32-channel body coil. Table 2 lists the MRI sequences and their parameters. An additional coronal fat-suppressed fast spin-echo T2-weighted image was obtained for a more precise evaluation and localization of the lesions.

### Imaging analyses

#### Evaluation of the RCC characteristics

All images were retrospectively and independently reviewed by radiologists S.T. and Y.U. (8 and 20 years of experience, respectively) who were unaware of all clinical and RNA information. A third radiologist (M.H., 23 years’ experience as a radiologist) helped reach a consensus when necessary (one case). The tumor diameter was measured in three planes on the CT image; the largest measurement was considered the tumor size (Table 1). The presence/absence of a pseudocapsule and that of fat in the tumor were determined. ’Pseudocapsule’ was defined as a thin, linear and regular hypointense band on T2WI surrounding the tumor, as described [24, 25]. The presence of fat was assessed by chemical shift dual-echo imaging.

For the tumor size evaluation explained below, we placed ROIs in the same way as that used for the perfusion maps. For each ROI, we calculated the signal/loss ratio (SLR) as: SLR = (SIin − SIout)/SIin, where SIin and SIout represent the signal intensity on in-phase and out-of-phase images, respectively. As described [26], when the maximum SLR value was >0.1, the tumor was considered to contain fat.

#### Measuring the perfusion parameters and ADCs of the RCCs

The perfusion parameters were measured on an Aquarius iNtuition client-server (TeraRecon, Durham, NC) workstation. To evaluate the relationships between the perfusion parameters and tumor size, we selected a slice on each tumor that appeared to be the largest and then placed as large an ROI as possible on the perfusion map. To evaluate the relationships between the perfusion parameters and RNA expression, we selected a slice based on a CT-guided biopsy image and then placed the ROI in the same range as the biopsy needle, based on the assumption that the RNA expression is the average value of the bulk region of the biopsy tissue. We ensured that the tumor ROI remained within the internal structure of the mass and excluded necrosis, cysts, hemorrhage, calcification, and adipose tissue.

We measured the ADCs by using a picture-archiving and communication system workstation (Synapse, FujiFilm, Tokyo). We selected the same slice on which the perfusion parameters were measured and placed two ROIs in the same way as was done for the perfusion parameters.

### Statistical analyses

We assessed the correlations between the tumor size, CCP score, and imaging parameters by determining Pearson’s product moment correlation coefficient. The imaging parameters were compared between the ccA and ccB groups using Student’s t-test. Fisher’s exact probability test was applied for the association between ccA/ccB subtype and tumor characteristics, e.g., the recognition of a pseudocapsule or the presence of fat. P-values <0.05 were considered significant. All statistical analyses were performed using R ver.3.5.1 (Vienna, Austria; http://www.R-project.org/).

## Results

**Fig 2:**
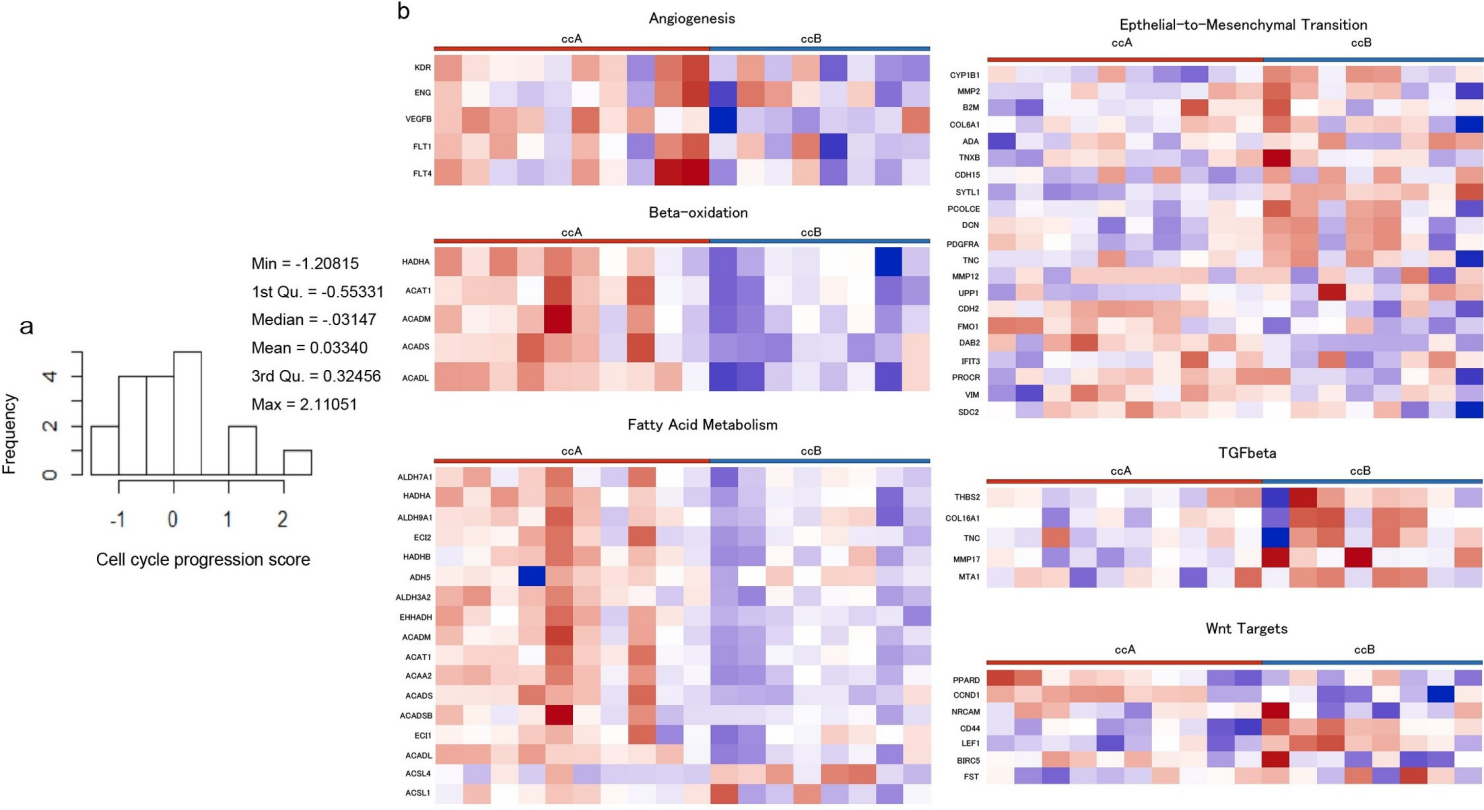
Tendencies in the transcriptome profiling. **a**: Distribution of all CCP scores. **b**: Results of the pathway analysis of subtypes showing that the ccA and ccB tumors are highly dissimilar, as previously reported [20]. Magnified heat maps of the genes that populate the ccA or ccB groups overexpressed MSigDB (Molecular Signatures Database)-curated gene sets of Brentani angiogenesis, beta-oxidation, fatty acid metabolism, EMT up, TGFβ C4 up, and Wnt targets.

Fig 2A shows the CCP scores. The mean CCP score was 0.033 (range: −1.20–2.11). Unsupervised clustering methods classified the ccA and ccB subtypes. Of 18 tumors, 10 were classified as ccA, and eight as ccB designation. A pathway analysis revealed that the better-prognosis ccA tumors showed relatively overexpressed genes that are associated with angiogenesis, the beta-oxidation pathway, and fatty acid metabolism. The ccB tumors overexpressed a more aggressive panel of genes that regulate the epithelial-mesenchymal transition (EMT), transforming growth factor-beta (TGF-β), and Wnt targets (Fig 2B). Table 1 includes a summary of the tumor characteristics. The mean tumor size was 24.8 mm (range: 14–40 mm). All of the perfusion parameters and the ADC could be measured, and the imaging parameters measured by the two ROI placement methods showed high correlations (Table 3).

**Table 3:** The correlations between ROIs for size and ROIs for RNA expression and the perfusion parameters and ADC.

Imaging parameters	ROIs for size	ROIs for RNA expression	rho	p-value
Kep, /min	3166 ± 1281 (1512–7225)	2231 ± 457 (1532–3071)	0.57	0.01
Ktrans, /min	873 ± 220 (576–1225)	868 ± 246 (485–1336)	0.84	<0.01
VP, ml/100 ml of tissue	17.3 ± 12 (3.0–50.7)	24.3 ± 17 (6.00–68.0)	0.83	<0.01
VE, ml/100 ml of tissue	370 ± 121 (183–619)	358 ± 122 (166–627)	0.82	<0.01
ADC, ×10^−3^ mm^2^/sec	1.36 ± 0.34 (0.63–1.99)	1.48 ± 0.27 (1.00–2.00)	0.93	<0.01

ADC: apparent diffusion coefficient, Ktrans: volume transfer constant, Kep: rate constant, VE: extracellular extravascular volume fraction, VP: fractional plasma volume.

### The relationships among tumor size, imaging parameters, and RNA expression

**Fig 3:**
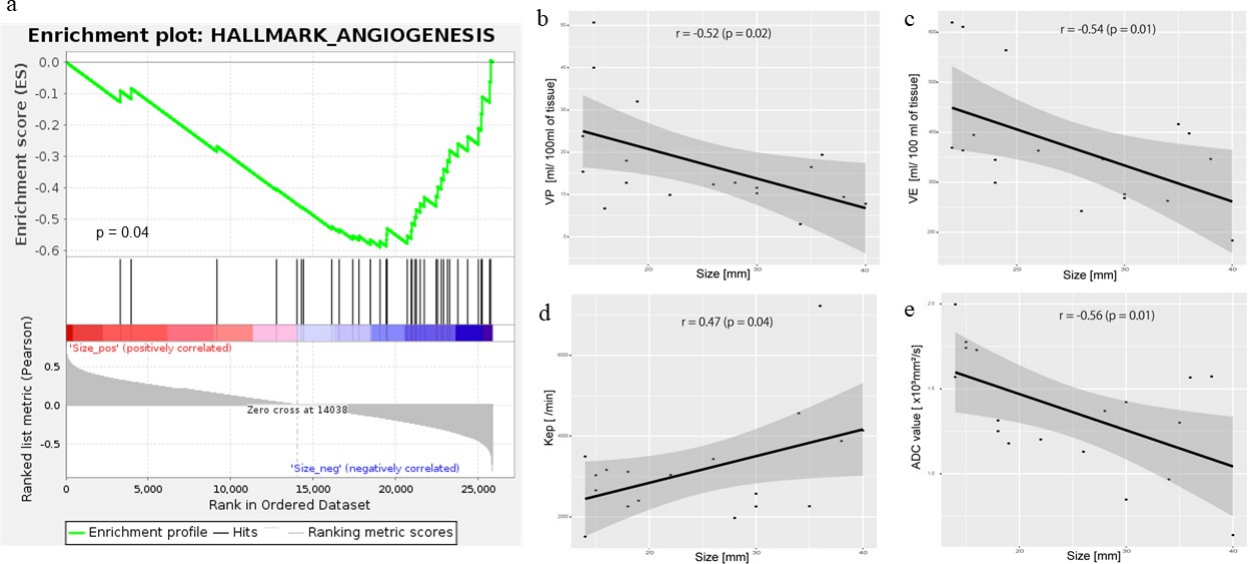
The relationships among tumor size, imaging parameters, and RNA expression. **a:** The GSEA revealed that decreased angiogenesis expression was significantly associated with increased tumor size (p = 0.041). **b:** The VP was inversely correlated with tumor size (r  =  −0.52, p = 0.02). **c:** The VE was inversely correlated with tumor size (r  =  −0.60, p = 0.01). **d:** The Kep was significantly correlated with tumor size (r = 0.47, p = 0.04). **e:** The ADC was inversely correlated with tumor size (r  =  −0.57, p = 0.01).

The enrichment analysis demonstrated only a significant inverse correlation between tumor size and angiogenesis RNA expression in the hallmark gene sets (p = 0.041) (Fig 3A). The other gene sets were not related to tumor size. In the pCT, tumor size was significantly correlated with Kep (p = 0.04) and inversely correlated with VE and VP (p = 0.02, 0.01) (Fig 3B–3D). In the MRI, tumor size was inversely correlated with the ADCs (p = 0.01) (Fig 3E). There was no relationship between tumor size and the CCP score (r = 0.17, p = 0.47) and no significant difference in the sizes of the ccA and ccB tumors (p = 0.80).

### The relationships between the CCP score and imaging parameters

**Fig 4:**
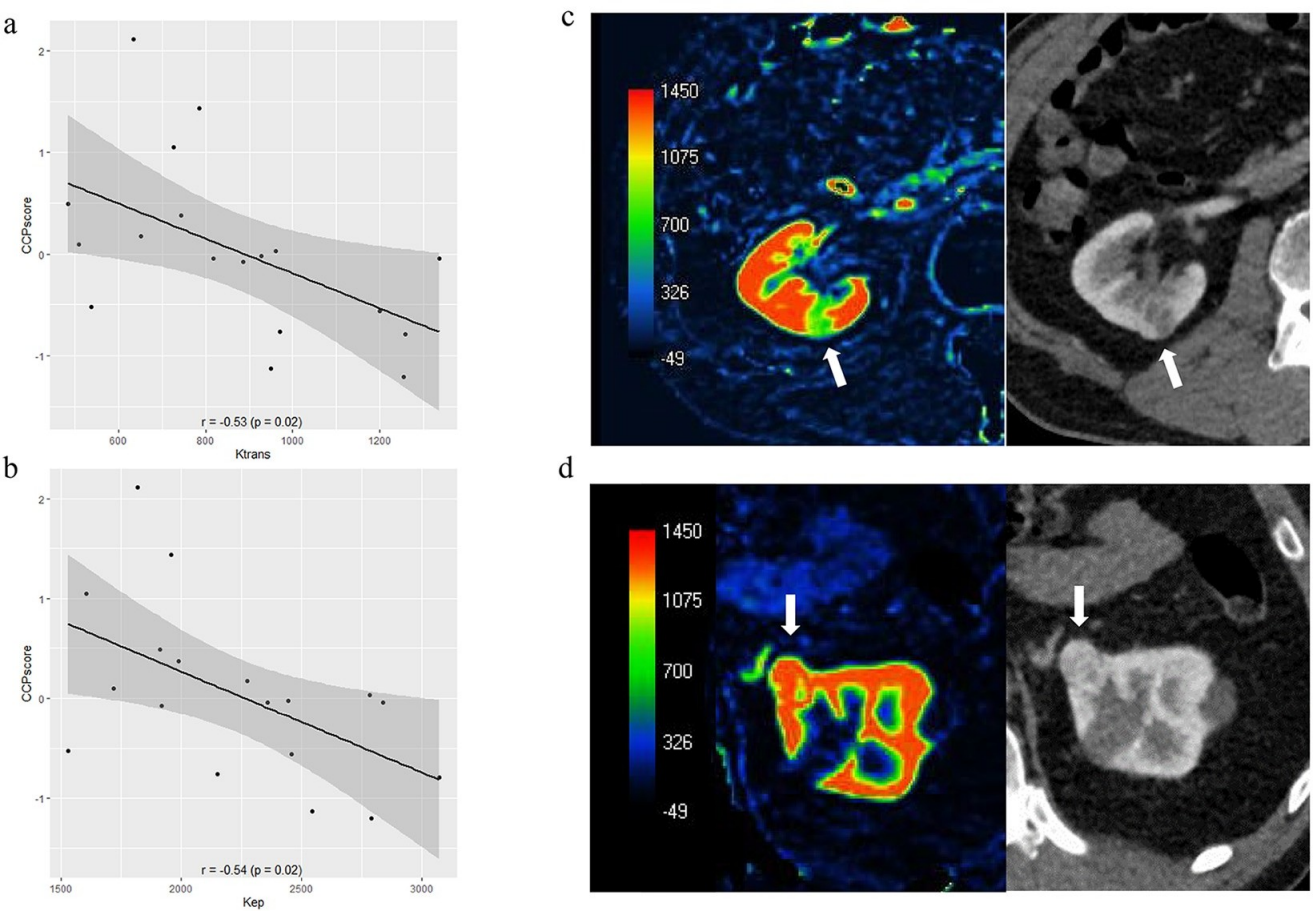
The relationships between the CCP score and the perfusion parameters. a: Ktrans was inversely correlated with the CCP score (r  =  −0.53, p = 0.02). b: Kep was inversely correlated with the CCP score (r  =  −0.54, p = 0.02). c,d: Two cases of clear cell carcinoma (arrow). They are the same size (18 mm), but the Ktrans value and the CCP score vary between them. c: The Ktrans is 509 and the CCP score is 0.09. d: The Ktrans is 1200 and the CCP score is −0.56.

Ktrans and Kep were inversely correlated with the CCP score (p = 0.02), and the tumors with high CCP scores tended to have lower Ktrans and Kep values (Fig 4).

### The relationships between the ccA/ccB subtype and the imaging parameters

**Fig 5:**
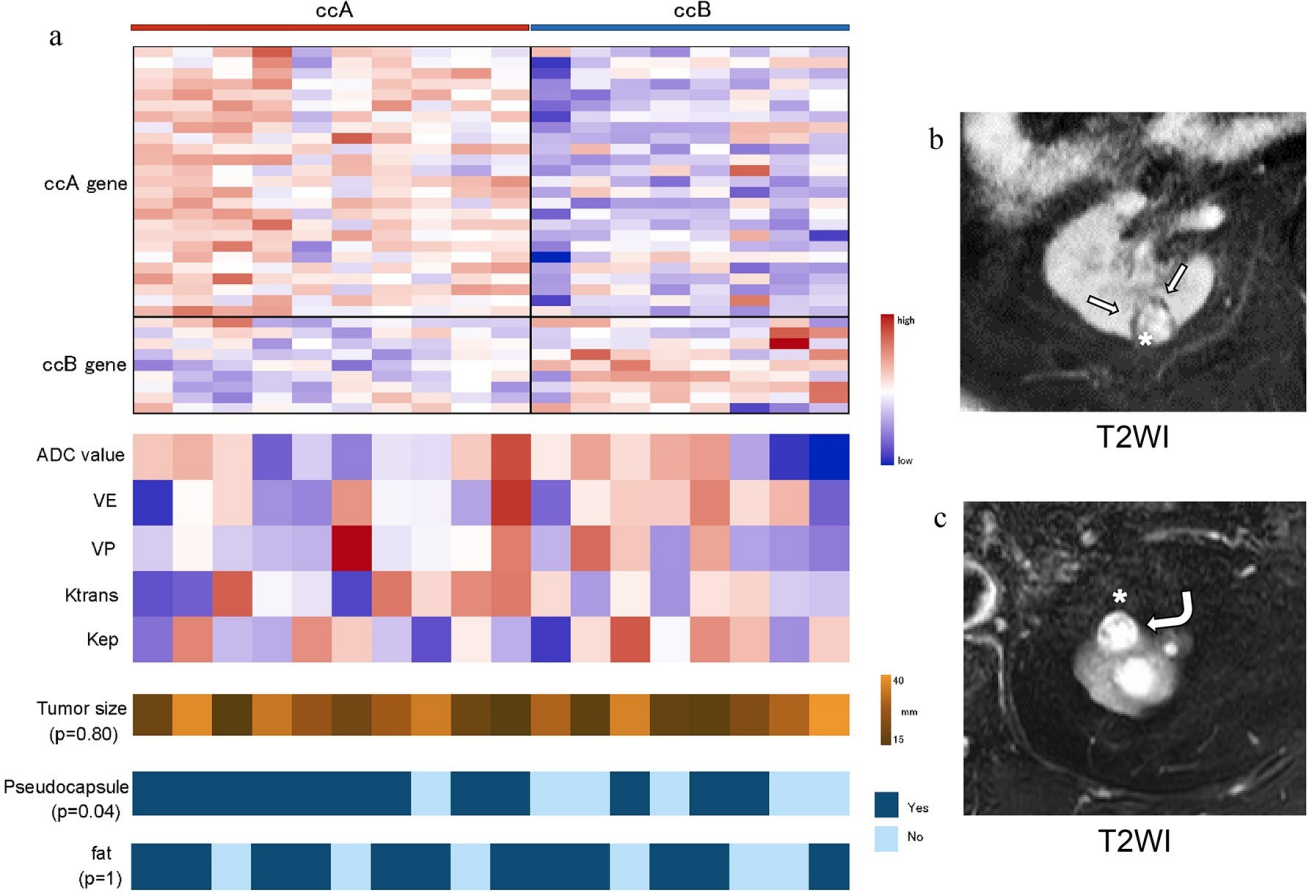
The relationships between the ccA/ccB subtype and the imaging parameters. **a:** The perfusion parameters, ADC, and tumor size were not correlated with the subtype. Only the presence/absence of a pseudocapsule (T2 rim) differed significantly between the subtypes (p = 0.04). **b, c:** Clear cell carcinomas (*asterisk*). **b:** MRI showed a hypointense rim surrounding the tumor in T2WI (*arrow*). This tumor was classified as the ccA subtype. **c:** MRI showed no hypointense rim in T2WI (*curved arrow*). This tumor was classified as the ccB subtype.

The tumors classified as ccA more frequently had a pseudocapsule compared to those classified as ccB (Fig 5). There were no significant differences in fat component, tumor size, or any of the imaging parameters between the ccA and ccB groups.

## Discussion

Our findings demonstrated that (1) the tumor size was significantly correlated with Kep and inversely correlated with VE, VP, the ADC, and angiogenesis RNA expression; (2) the CCP score was inversely correlated with Kep and Ktrans, and (3) a pseudocapsule tended to be recognized in the tumors classified as ccA. To our knowledge, this is the first study to demonstrate relationships between imaging results and RNA expression data in RCC.

**Fig 6:**
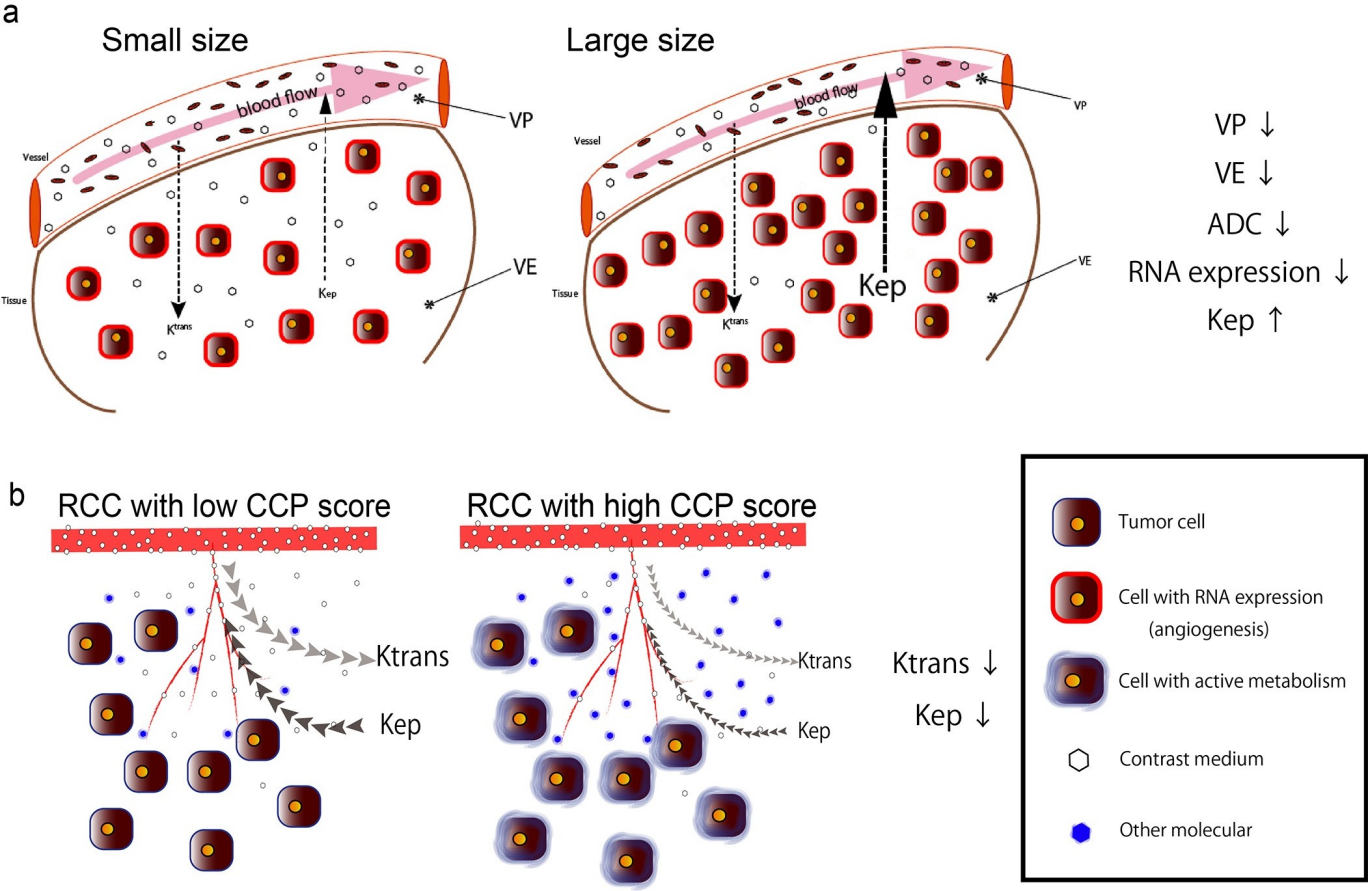
Consideration of the molecular pathogenesis in small RCC. **a:** Schema of the relationships among the tumor size, RNA expression, and imaging parameters. Low perfusion and increased cell density occur with tumor growth. **b:** Schema of the relationships between the CCP score and the perfusion parameters. RCCs with a high CCP score developed a more aggressive tumor microenvironment, resulting in reduced permeabilities.

We observed that the tumor size (used as a risk factor in surveillance) was inversely correlated with the VE, VP, and ADC. This suggests that the number of cells increases and the blood flow decreases with increasing tumor volume. This is consistent with the report of an inverse relationship between tumor diameter and microvascular density [27]. The positive relationship between Kep and tumor size indicates that the rate of contrast flow from tumor tissue to plasma increases with the tumor size. We also observed that the angiogenesis RNA expression level decreased with increasing tumor size. These relationships between tumor size and each parameter indicate that the internal pathology of small RCC changes dynamically with the tumor’s growth; i.e., the cell density is increased, resulting in a hypoperfusion status as the tumor increases in size (Fig 6a).

A Hypoperfusion status may result in tumor hypoxia, which has been implicated in tumor propagation [28]. Our present findings thus suggest that tumor-size monitoring has some utility for predicting the grade in the current surveillance regimens. However, tumor size was not associated with the CCP score or the ccA/ccB subtype as a risk predictor for prognosis or metastasis herein; i.e., the tumor size is not a sufficiently accurate risk factor for small RCC.

The CCP score is calculated using a set of genes involved in the cell cycle, and it reflects the percentage of actively dividing cells in the tissue [29]. The CCP score was reported to have value for predicting metastatic progression [17] and is also a significant, independent predictor of long-term oncologic outcomes in RCC [18]. Our findings indicate that RCCs with higher CCP scores have lower rates of iodine contrast flow from the plasma to the extracellular space and from the extracellular space to the plasma, but there was no correlation between the ADCs and the CCP scores (r  =  −0.3, p = 0.18). This suggests that RCCs with high CCP scores may develop a more aggressive tumor microenvironment and have higher concentrations of various metabolites and cytokines compared to RCCs with low CCP scores, resulting in a lower iodine distribution in the tumor’s extracellular space (Fig 6B). The use of sorafenib in RCC showed good progression-free survival (PFS) in tumors with high Ktrans values [30, 31]. Therefore, RCCs with high Ktrans values could have low CCP scores and lower cell proliferation levels, which offer better PFS based on our present results.

In our evaluation of the relationships between the ccA/ccB subtype and imaging parameters, all of the RCCs could be classified as ccA or ccB, and the pathway analysis (Fig 2B) gave results that are similar to reported values [20]. RCCs classified as ccA have better prognoses than those classified as ccB, and the ccA/ccB classification has been applied to large cohorts of patients with metastatic disease, demonstrating the classification’s utility for predicting prognoses and the response to sunitinib [32, 33]. Herein, a pseudocapsule could be identified in the ccA-subtype tumors. It was shown that RCCs with a pseudocapsule were histologically low-grade [34]. Taken together, the previous and present results suggest that small RCCs with an identified pseudocapsule may have better prognoses than those in which a pseudocapsule could not be identified.

We detected no association between the CCP score and the ccA/ccB subtypes. In general, some association between predictors of metastasis and predictors of poor prognosis is assumed. Our result may be attributed to the small sample size as discussed below, or the nature of RCCs which show slow growth.

Several study limitations should be addressed. The RNA expression was evaluated by a single biopsy, and we did not assess tumor heterogeneity. However, RNA expression, especially regarding the CCP score and ccA/ccB subgroup, is less heterogeneous in small RCC [6]. The ROIs for imaging parameters for the comparison with RNA expression were placed along the biopsy area, and they correlated strongly with the ROIs at the largest section of the tumor (Table 2). It therefore seems appropriate to evaluate the RNA expression using single-biopsy data because the tumor heterogeneity was assessed in terms of both RNA expression and imaging.

Normal tissue may have been present within the biopsy tissue; however, we confirmed that the biopsy needle was always in the RCC under CT guidance, and every biopsy tissue sample was cytologically confirmed for atypical cells. There was thus a low probability that contamination occurred. A third limitation is that minimally invasive treatments for small RCC (e.g., partial nephrectomy and cryotherapy) are already established. However, it is not clinically effective or cost-effective to treat all small RCCs because the median age at the diagnosis of RCC is 64 years, and many of these patients have significant competitive comorbidities. Moreover, the relatively stable mortality rate of RCC, despite its increased incidence, has suggested overdiagnosis and overtreatment [35]. We thus suggest that there is a lack of risk stratification for RCC. Finally, our cohort was small (n = 18). Further study is thus warranted. This preliminary study is currently being extended to a larger population.

In conclusion, tumor size was correlated with low perfusion, but tumor size is not a sufficient risk factor to identify agressive small RCCs based on genomic profiling. By contrast, Kep, Ktrans, and the recognition of a pseudocapsule were associated with RNA expression as poor prognostic factors. Our quantitative and qualitative assessment results may provide a valid risk stratification of small RCCs based on RNA expression, and they may provide guidance regarding the timing and duration of appropriate therapeutic interventions and follow-up periods for patients with small RCCs.

## Supporting information

S1 Table Details of the tumor diameters and imaging parameters to evaluate the relationship with size. (DOCX)

S2 Table Details of CCP scores, tumor subtypes and imaging parameters to evaluate the relationship with RNA expression. (DOCX)

## Abbreviations

ADC: apparent diffusion coefficient
AS: active surveillance
ccA: clear cell type A
ccB: clear cell type B
CCP: cell-cycle progression
DWI: diffusion-weighted imaging
GSEA: gene set enrichment analysis
pCT: perfusion computed tomography
RCC: renal cell carcinoma
RNA: ribonucleic acid
