# Supplementary material for: Radiology- and gene-based risk stratification in small renal cell carcinoma: A preliminary study
Source: PLoS One. 2021 Sep 7;16(9):e0256471. doi: 10.1371/journal.pone.0256471 (PMC8423232; doi:10.1371/journal.pone.0256471)
Supplement: S1 Table — (DOCX) [file pone.0256471.s001.docx]

**S1 Table. Details of the tumor diameters and imaging parameters to evaluate the relationship with size.**

| No | Tumor dia., mm | Kep, /min | Ktrans, /min | VP, ml/100 ml of tissue | VE, ml/100 ml of tissue | ADC, ×10^−3^ mm^2^/sec |
| --- | --- | --- | --- | --- | --- | --- |
| rcc00013 | 18 | 2259.5 | 585.09 | 12.78 | 344.9 | 2022 |
| rcc00014 | 30 | 2574.3 | 576.1 | 11.6 | 268.3 | 1619 |
| rcc00016 | 38 | 3880.7 | 1135.4 | 9.4 | 346.6 | 1834 |
| rcc00017 | 15 | 3030.5 | 887.1 | 50.7 | 363.9 | 1758 |
| rcc00021 | 14 | 3496.0 | 980.2 | 15.4 | 369.1 | 1595 |
| rcc00024 | 34 | 4566.8 | 615.7 | 3.0 | 262.7 | 1302 |
| rcc00026 | 36 | 7224.8 | 1225.2 | 19.4 | 397.8 | 1567 |
| rcc00027 | 26 | 3432.2 | 786.5 | 12.4 | 242.4 | 1580 |
| rcc00029 | 16 | 3163.8 | 1078.55 | 6.7 | 394.7 | 1729 |
| rcc00032 | 19 | 2401.4 | 1045.2 | 32.0 | 564.1 | 1224 |
| rcc00034 | 15 | 2658.4 | 1171.8 | 40.0 | 611.0 | 1776 |
| rcc00035 | 28 | 1974.0 | 659.6 | 12.8 | 345.9 | 1422 |
| rcc00037 | 35 | 2263.7 | 851.8 | 16.5 | 416.2 | 1468 |
| rcc00038 | 22 | 3039.2 | 1129.3 | 9.9 | 363.3 | 1350 |
| rcc00039 | 18 | 3117.4 | 855.2 | 18.0 | 299.1 | 1941 |
| rcc00040 | 14 | 1512.3 | 860.7 | 23.8 | 619.7 | 2067 |
| rcc00041 | 30 | 2257.6 | 614.2 | 10.3 | 276.0 | 1251 |
| rcc00043 | 40 | 4140.1 | 672.5 | 7.81 | 183.6 | 1386 |

ADC: apparent diffusion coefficient, Ktrans: volume transfer constant, Kep: rate constant, VE: extracellular extravascular volume fraction, VP: fractional plasma volume
