# Supplementary material for: Radiology- and gene-based risk stratification in small renal cell carcinoma: A preliminary study
Source: PLoS One. 2021 Sep 7;16(9):e0256471. doi: 10.1371/journal.pone.0256471 (PMC8423232; doi:10.1371/journal.pone.0256471)
Supplement: S2 Table — (DOCX) [file pone.0256471.s002.docx]

**S2 Table. Details of CCP scores, tumor subtypes and imaging parameters to evaluate the relationship with RNA expression.**

| No | CCP score | Tumor subtype | Kep, /min | Ktrans, /min | VP, ml/100 ml of tissue | VE, ml/100 ml of tissue | ADC, ×10^−3^ mm^2^/sec |
| --- | --- | --- | --- | --- | --- | --- | --- |
| rcc00013 | 0.097105 | CcA | 1718 | 509 | 16 | 166 | 1636.5 |
| rcc00014 | -0.52518 | CcB | 1532 | 538 | 13 | 213 | 1520.5 |
| rcc00016 | -0.04099 | CcA | 2838 | 1336 | 24 | 349 | 1703.5 |
| rcc00017 | -0.04428 | CcB | 2358 | 818 | 53 | 372 | 1750 |
| rcc00021 | 1.435723 | CcA | 1960 | 786 | 16 | 401 | 1582.5 |
| rcc00024 | 0.490548 | CcA | 1911 | 485 | 14 | 249 | 1134.5 |
| rcc00026 | -0.78955 | CcB | 3071 | 1259 | 34 | 417 | 1567 |
| rcc00027 | 0.030937 | CcA | 2783 | 962 | 13 | 239 | 1355 |
| rcc00029 | -0.76234 | CcB | 2150 | 970 | 9 | 416 | 1729 |
| rcc00032 | -0.56269 | CcA | 2458 | 1200 | 68 | 498 | 1201.5 |
| rcc00034 | -1.20815 | CcB | 2791 | 1255 | 41 | 522 | 1776 |
| rcc00035 | 0.375562 | CcA | 1989 | 743 | 19 | 334 | 1396 |
| rcc00037 | 1.051881 | CcA | 1605 | 727 | 21 | 331 | 1384.5 |
| rcc00038 | -1.13194 | CcB | 2544 | 950 | 11 | 394 | 1276 |
| rcc00039 | 0.171559 | CcA | 2275 | 651 | 23 | 266 | 1627.5 |
| rcc00040 | -0.07548 | CcA | 1918 | 886 | 49 | 627 | 2032.5 |
| rcc00041 | 2.110514 | CcB | 1818 | 634 | 9 | 448 | 1049.5 |
| rcc00043 | -0.02195 | CcB | 2444 | 928 | 6 | 206 | 1012.5 |

ADC: apparent diffusion coefficient, CcA: clear cell type A, CcB: clear cell type B, CCP: cell-cycle progression, Ktrans: volume transfer constant, Kep: rate constant, VE: extracellular extravascular volume fraction, VP: fractional plasma volume
